# Acceptance and Expectations of Medical Experts, Students, and Patients Toward Electronic Mental Health Apps: Cross-Sectional Quantitative and Qualitative Survey Study

**DOI:** 10.2196/14018

**Published:** 2019-11-25

**Authors:** Gwendolyn Mayer, Nadine Gronewold, Simone Alvarez, Bastian Bruns, Thomas Hilbel, Jobst-Hendrik Schultz

**Affiliations:** 1 Department of General Internal Medicine and Psychosomatics Heidelberg University Hospital Heidelberg Germany; 2 Medical Faculty of Heidelberg Heidelberg University Heidelberg Germany; 3 Electrical Engineering and Applied Sciences Westphalian University of Applied Sciences Gelsenkirchen Germany

**Keywords:** acceptance, telemedicine, telehealth, eHealth, mHealth, interventions, depression, patients, students, experts, expectation, risk

## Abstract

**Background:**

The acceptability of electronic mental (e-mental) health apps has already been studied. However, the attitudes of medical experts, students, and patients taking into account their knowledge of and previous experiences with e-mental health apps have not been investigated.

**Objective:**

The aim of this study was to explore the attitudes, expectations, and concerns of medical experts, including physicians, psychotherapists and nursing staff, students of medicine or psychology, and patients toward e-mental health apps when considering their knowledge of and former experiences with e-mental health apps.

**Methods:**

This cross-sectional quantitative and qualitative survey was based on a self-developed questionnaire. A total of 269 participants were included (104 experts, 80 students, and 85 patients), and 124 eligible participants answered a paper version and 145 answered an identical online version of the questionnaire. The measures focused on existing knowledge of and experiences with e-mental health apps, followed by a question on whether electronic health development was generally accepted or disliked. Further, we asked about the expectations for an ideal e-mental health app and possible concerns felt by the participants. All items were either presented on a 5-point Likert scale or as multiple-choice questions. Additionally, 4 items were presented as open text fields.

**Results:**

Although 33.7% (35/104) of the experts, 15.0% (12/80) of the students, and 41.2% (35/85) of the patients knew at least one e-mental health app, few had already tried one (9/104 experts [8.7%], 1/80 students [1.3%], 22/85 patients [25.9%]). There were more advocates than skeptics in each group (advocates: 71/104 experts [68.3%], 50/80 students [62.5%], 46/85 patients [54.1%]; skeptics: 31/104 experts [29.8%], 20/80 students [25.0%], 26/85 patients [30.6%]). The experts, in particular, believed, that e-mental health apps will gain importance in the future (mean 1.08, SD 0.68; 95% CI 0.94-1.21). When asked about potential risks, all groups reported slight concerns regarding data security (mean 0.85, SD 1.09; 95% CI 0.72-0.98). Patient age was associated with several attitudes toward e-mental health apps (future expectations: r=–0.31, *P*=.005; total risk score: r=0.22, *P*=.05). Attitudes toward e-mental health apps correlated negatively with the professional experience of the experts (r_s_(94)=–0.23, *P*=.03).

**Conclusions:**

As opposed to patients, medical experts and students lack knowledge of and experience with e-mental health apps. If present, the experiences were assessed positively. However, experts show a more open-minded attitude with less fear of risks. Although some risks were perceived regarding data security, the attitudes and expectations of all groups were rather positive. Older patients and medical experts with long professional experience tend to express more skepticism.

**Trial Registration:**

German Clinical Trials Register DRKS00013095; https://www.drks.de/drks_web/navigate.do? navigationId=trial.HTML&TRIAL_ID=DRKS00013095

## Introduction

### Background

Smartphone apps for mental disorders, so called electronic mental (e-mental) health apps, have the potential to deliver immediate therapeutic help for various illnesses like substance abuse, bipolar disorders, depression, anxiety, psychosis, and even suicide [1]. They offer support at any time and place and provide context-aware interventions and real-time feedback. A recent review found 165 primary research studies on smartphone interventions for mental health in 2017-2018, and much evidence has been provided according to the effectiveness of these interventions [2]. In particular, patients suffering from depression benefit from e-mental health apps [3], and some clinical experts have welcomed this development to empower patients toward improved care [4].

Nevertheless, e-mental health apps available in the app stores of the market leaders Google and Apple are rarely clinically validated, and only a few are registered under the European Regulation on medical devices [5]. Thus, it is hardly surprising that there is criticism about the potential adverse effects such as low quality of therapeutic content or replacement of health care contacts [6]. In fact, the real-world user engagement of e-mental health apps beyond the clinical setting is rather low. There is a high attrition rate due to drop outs after a few days or weeks of use [7]. Recently, Fleming and Bavin [8] showed that the completion or sustained use of these programs varied from 0.5% to 28.6% [8]. One of the reasons for this gap between clinical trials and real-world engagement lies in different target populations of trials and people using apps in the real world [9]. However, some authors found that low usability, concerns about privacy, and a lack of trust prevent potential users to create the necessary confidence in e-mental health apps [10].

To bridge this gap and make clinically valid, effective interventions available to the broad public, a deeper understanding of the attitudes of all stakeholders is necessary. These are first and foremost patients in clinical and outpatient settings, medical experts who are in close contact with the psychotherapeutic process (such as physicians, psychological and medical psychotherapists, and nursing staff) and finally, future professionals who are current students of medicine or psychology.

#### Attitudes of Patients

Previous studies on attitudes toward e-mental health apps observed that the majority of the participants prefer face-to-face therapy over Web-based interventions. Interestingly, anonymity is the least important concern for rejecting e-mental health apps, while helpfulness, credibility, and accessibility are more important [11]. Research on privacy concerns reveals an inconsistent picture: The intention of patients to share personal health information with health care providers, in general, is highly influenced by privacy concerns [12]. This may result in a lower willingness to trust e-mental health apps, which of course implies sharing intimate experiences with software of unknown origin. In qualitative interviews, patients have expressed concerns about becoming dependent on apps or of losing social support [13].

Despite many doubts, patients also see advantages in using apps targeting mental disorders. These possibilities range from the acquisition of new skills, social connectedness, and feelings of a “safety netting” [13] to a deeper understanding of personal mood and triggers of their mental health problems [14] and even alarm functions and reminders for clinical appointments for patients with psychosis [15]. Internet interventions, in general, were rated as helpful, while guided programs or videoconferencing were preferred over unguided self-help programs [16]. Recent results show that especially patients with negative care experiences tend to prefer electronic health (eHealth) services, in particular, those with lower educational levels [17].

The relationship with physicians also seems to play a role in the acceptance of e-mental health apps for patients. A strong “doctor-related locus of control” has been negatively associated with the intention to use e-mental health apps [18]. Similarly, the willingness of patients to use e-mental health apps and programs depends to a high degree on their acceptance by the respective clinician [19] and even on the awareness of experts in teaching related topics to their medical students [20].

#### Attitudes of Medical Experts

Physicians in Germany show a positive attitude toward future eHealth developments, in general; nonetheless, some voice concerns about immature technology and neglected privacy [21]. However, only half of the established physicians in Germany feel adequately informed about these developments [22].

What expectations of and knowledge about e-mental health apps do physicians really have? A glance at topics in American mental health–related conferences in 2013-2015 shows that only 0.3% of the sessions addressed e-mental health apps [20]. This number, of course, may be higher today. A closer look reveals the underlying divided opinions of medical experts about digital health interventions. On the one hand, mental health care staff fear that internet-based services could replace face-to-face support. On the other hand, access to helpful information at any time and place, the possibility to express oneself in forums, and the incorporation of psychoeducational material is perceived as a great asset. Finally, internet-based services have been seen to possibly lower the threshold to initiate psychotherapy [23]. One recent study, which specifically investigated the attitudes of physicians and psychotherapists, showed that experts doubt the possibility of effective treatment via the internet, but they regard telemedicine as a possible potential supplement to conventional face-to-face therapy [24]. A direct comparison of the attitudes of patients with depression and psychotherapists found more negative attitudes among psychotherapists than patients. Similarly, patients in clinical settings seemed to be more skeptical than patients recruited via the internet [25].

#### Missing Aspects

Three important aspects of attitudes toward e-mental health are missing in the studies highlighted above. First, the opinion of the future medical experts, that is, students of medicine or psychology, has not been studied. Apart from the integration of eHealth topics in single universities in the medical curriculum [26,27] and the willingness to use mental health apps by students themselves [28,29], data on the attitudes of students toward the topic are limited.

Second, the attitudes and expectations of the nursing staff, who are in close contact with patients who have used mental health apps or plan to do so, need to be considered. A study that inquired about their views on eHealth development, in general, not specific to e-mental health apps, reported that staff fear a loss of quality in social interaction caused by care robots while benefiting from process improvements due to digital documentation systems [22]. However, their opinions specifically about e-mental health apps are missing.

Third, and probably most important, none of the studies have asked the participants about their previous knowledge and personal experiences with e-mental health apps. It is conceivable that this contributes to the insights delivered by the research on user engagement mentioned in the beginning. Despite a growing amount of evidence-based interventions available on e-mental health apps, the dropout rates are high. This has been explained by poor usability, lack of trust, and concerns about missing data security [10]. The following question arises: Have the reservations about these tools increased or decreased depending on existing knowledge and personal experiences with e-mental health apps?

In this study, we have integrated these missing aspects in our research design to contribute to a deeper understanding of the barriers and facilitators of real-world engagement of e-mental health apps.

### Objective

This study investigated the attitudes, expectations, and concerns toward e-mental health apps of physicians, psychological psychotherapists, psychotherapists in training, nurses, students of medicine or psychology, and patients with relevant support needs who could be prospective users of e-mental health apps.

The main objectives were based on the following research questions: How many e-mental health apps do the participants know of? How many have they tried and what were their experiences? Are e-mental health apps accepted or disliked, and for what reasons? What expectations do these groups have and what risks do they see?

Finally, the study also reports on the possible determinants of attitudes like age and sex of all groups as well as the number of years of professional experience of the medical experts.

## Methods

### Study Design

We conducted a cross-sectional quantitative and qualitative study using a questionnaire that was designed for the purpose of this survey. Data were collected between September 2017 and June 2018. The questionnaire was distributed either as a Web-based version via the online tool SoSci Survey [30] or in a paper-pencil version. We obtained a sample of 269 participants; 124 eligible participants answered the paper version and 145 answered the online version.

The study “Acceptance and expectations of experts, students and patients according to health apps for mental disorders” was registered in the German Clinical Trials Register (DRKS00013095).

### Target Population and Recruitment

This study focused on physicians in the disciplines of General Medicine, Internal Medicine, Psychosomatics, Psychiatry, or Psychotherapy as well as psychological psychotherapists, trainee psychotherapists and nurses, students of medicine or psychology, and patients with a psychosomatic disorder.

Recruitment was performed with postings and mail distribution services to medical experts and students within the University Hospital of Heidelberg’s Department of Internal Medicine and Psychosomatics in the Medical Faculty and in the Psychological Institute of the University of Heidelberg. More than 120 patients of the inpatient and outpatient services in the Department of Internal Medicine were approached with the paper-pencil version. Further participants were recruited via the internet. We wrote more than 800 personalized emails to physicians and psychotherapists published by the Association of Statutory Health Insurance Physicians. Further, students and patients received an invitation via Facebook, XING, LinkedIn, SurveyCircle, deutsche depressionsliga, and Diskussionsforum Depression. All groups and forums gave their consent in advance. Patients were excluded if they stated that they did not have a mental disorder or another disease that affects mental health (in the Web version only). No reward was given for participation in the study.

Ethical approval was obtained by the Ethics Commission of the Medical Faculty of Heidelberg (S336-2017) prior to data collection.

### Sample Description

We collected 285 completed questionnaires. We then excluded 16 questionnaires; in one case, we received no informed consent, and 15 participants did not fulfill the inclusion criteria.

The sample consisted of 269 participants aged between 18 and 77 years (mean 37.39 years, SD 14.14 years). The demographic characteristics of the sample are presented in Table 1. Nearly two-thirds were female (173/269 [64.3%]). The sample comprised 104 medical experts, 80 students, and 85 patients. The questionnaire was filled in by 43 physicians, 33 psychological psychotherapists, 16 psychotherapists in training, and 13 nurses. As one physician was also a psychotherapist in training, there were 104 experts in total. Of the 80 students, 54 were students of medicine and 28 were students of psychology (two of them studied both). Finally, 41 patients were recruited from the University Hospital of Heidelberg (Internal Medicine), and 44 patients were recruited via the internet.

**Table 1:** Descriptive statistics of the study sample (N=269).

Characteristics		Participants, n (%)
Age (years), mean (SD)		37.39 (14.14)
**Sex**		
	Female	173 (64.31)
	Male	96 (35.69)
	Other	0 (0)
**Nationality**		
	German	251 (93.31)
	Other	18 (0.69)
**Family status**		
	Single	158 (58.74)
	Married	86 (31.97)
	Separated	4 (1.49)
	Divorced	14 (5.20)
	Widowed	1 (0.37)
	Other	5 (1.86)
	Missing	1 (0.37)
**Profession (experts)^a^**		
	General medical practitioner	7 (6.73)
	Specialist in internal medicine	23 (22.12)
	Specialist in psychosomatics	12 (11.54)
	Specialist in psychiatry	9 (8.65)
	Specialist in psychotherapy	6 (5.77)
	Physician (total)	43 (41.35)
	Psychological psychotherapist	33 (31.73)
	Psychotherapist in training	16 (15.38)
	Nurse	13 (12.50)
	Experts (total)	104 (100)
**Subject (students)^a^**		
	Medicine	54 (67.50)
	Psychology	28 (35.00)
	Students (total)	80 (100)
**Patients**		
	Patient of the University Hospital Heidelberg	41 (48.24)
	Patient recruited via the internet	44 (51.76)
	Patients (total)	85 (100)
**Education (patients)^a^**		
	Still in school	1 (1.18)
	Secondary school	13 (15.29)
	Secondary high school	24 (28.24)
	Higher school certificate	26 (30.59)
	Study exam	18 (21.18)
	Other	3 (3.53)

^a^Multiple choice possible.

### Measures

We developed a 10-minute, structured questionnaire that consisted of two major parts: The first section was titled “Demographics” and contained nine items; the second section was titled “Attitudes towards e-mental health apps” and contained 25 items. We distributed three versions of the questionnaire to the various target groups: medical experts, students, and patients. The versions differed slightly regarding the first part.

The sociodemographic data obtained were age, sex, nationality, marital status, socioeconomic status, and either profession or study subject. The medical experts were further asked to state their professional experience in years.

The items of the section “Attitudes towards e-mental health apps,” the results of which are reported in this article, are listed in Table 2 and sorted by issues in the same order presented in the questionnaire.

The first part of the section “Attitudes towards e-mental health apps” addressed individual knowledge and prior experiences with the most common e-mental health apps at the time of the survey. If present, the experiences could be rated with a 5-point Likert scale from “negative” to “positive.” After that, the questions asked for attitudes toward e-mental health apps, in general. The participants were asked to choose one option of five statements, from “I am concerned about the development” to “I think, there’s great potential in the development.” A further item asked for the participant’s opinion about whether e-mental health apps will gain importance in the future, with a 5-point Likert scale ranging from “no” to “yes.”

The next section of the questionnaire asked for expectations toward an ideal e-mental health app in eight statements (ie, “Privacy should be respected” or “The design should be appealing”). Another part asked for four different risks referring to no helpfulness, harmfulness to health, loss of social contacts, and lack of data security. In this section, the Cronbach alpha was .56 as well. Both parts, expectations and risks, were presented as a 5-point Likert scale from “not important” to “very important” and “no risk” to “high risk,” respectively.

Additionally, four items were presented as open-text fields in order to give the participants the opportunity to express their opinions in a more detailed way.

**Table 2:** Items of the section “Attitudes towards e-mental health apps” sorted by issues.

Issue and item			Type
**Knowledge**			
	Which of the following apps do you know? (list)	Multiple choice	
**Experiences**			
	Which of the following apps did you already try? (list)	Multiple choice	
	How do you rate your experiences?	5-point Likert scale^a^	
**Attitudes (1)**			
	What do you think in general of e-mental health apps?	Rank order (ordinal)	
**Attitudes (2)**			
	Do you think, that e-mental health apps will gain importance in the future?	5-point Likert scale^a^	
**Positive aspects**			
	Which positive aspects do you see regarding e-mental health apps?	Open-text field	
**Expectations toward an ideal e-mental health app**			
	What functions or properties would you like to have in an ideal e-mental health app? (8 sub items)	5-point Likert scale^a^	
	Further functions or properties	Open-text field	
**Negative aspects**			
	What would stop you from using an e-mental health app?	Open-text field	
**Risks**			
	What risks do you see in e-mental health apps? (4 sub items)	5-point Likert scale^a^	
	Further risks	Open-text field	

^a^Range: –2 to +2.

### Statistical Analysis

Data analysis was conducted using SPSS Statistics (version 24; IBM Corp, Armonk, New York) [31]. The data of the three groups were recorded simultaneously and analyzed for group differences. The preliminary exploration of the descriptive statistics was performed by calculating frequencies, means and SDs, and reporting 95% CIs. The range of all continuous items was coded from –2 to +2.

We explored differences between the three groups in attitudes by using the Pearson chi-square tests for attitudes rated on an ordinal scale and by using a 1-way analysis of variance (ANOVA) for attitudes rated on a 5-point Likert scale. In case of variance homogeneity, we calculated post-hoc tests according to Scheffé; if variance homogeneity was missing, we chose Dunnett-T3 due to its ability to discover even small differences among groups [32].

To explore the expectations toward an ideal e-mental health app, we carried out descriptive measures and an additional factor analysis (root cause analysis) in order to identify main components of the expectations toward an ideal e-mental health app and conducted a further ANOVA with the factors. The Cronbach alpha was calculated to assess the internal consistency of this section. It was rather low with an α=.56, which could be due to the heterogeneity of the concept [33].

The risks seen by the three groups were calculated via descriptive measures and an ANOVA. Additionally, we calculated a total risk score by averaging the four risks.

Looking for possible determinants of attitudes, expectations, and risks, we calculated correlations for age and professional experience of the experts. When appropriate, we calculated Pearson correlations (*r*) and Spearman rank correlations (*r_s_*). We looked for sex differences by calculating the Mann-Whitney *U* tests or an ANOVA, depending on the scale levels of the items.

The qualitative data in open-text fields were analyzed manually by building inductive categories and taking into account the recommendations of content analysis and its possible quantification of categories following Mayring [34].

## Results

### Knowledge and Previous Experiences With Electronic Mental Health Apps

Of a short list of common e-mental health apps, 33.7% (35/104) of the experts and 15.0% (12/80) of the students indicated that they knew at least one of the apps (Table 3). The percentage of experts and students who had already tried one e-mental health app was 8.7% (9/104) and 1.3% (1/80), respectively. In the group of the patients, 41.2% (35/85) knew at least one app and 25.9% (22/85) had at least tried one app. The patients who had already tried at least one app were patients of the University Hospital of Heidelberg in three cases, and the other 19 were recruited via the internet.

When prior experiences with an e-mental health app were present, they were evaluated as positive. The nine experts who stated to already have experiences with an app rated the experiences with a mean of 1.22 (SD 0.44, 95% CI 0.88-1.56). The 22 patients rated their experiences with a mean of 1.18 (SD 0.96, 95% CI 0.76-1.61).

**Table 3:** Results for the question, “Which of the following apps do you know?”

App	Experts (n=54), n	Students (n=16), n	Patients (n=58), n	Total (N=128), n (%)
ARYA	2	1	4	7 (5.47)
DepressionsCoach (TK)	14	4	9	27 (21.0)
Deprexis24	16	0	3	19 (14.84)
Human Progress	0	3	2	5 (3.91)
Meplus	0	0	1	1 (0.78)
Minddistrict	2	0	2	4 (3.13)
Moodgym	5	0	4	9 (7.03)
Moodpath	1	2	11	14 (10.94)
MyTherapy	2	4	7	13 (10.16)
Novego	0	0	1	1 (0.78)
Selfapy	5	2	3	10 (7.81)
Others	7	0	11	18 (14.10)

### Attitudes Toward Electronic Mental Health Apps

#### Quantitative Results

There were more proponents than skeptics against e-mental health apps (Table 4): 28.6% answered skeptically by choosing one of the first two options (31/104 [29.8%] experts, 20/80 [25.0%] students, 26/85 [30.6%] patients), 62.1% showed positive attitudes by choosing one of the last two options (71/104 [68.3%] experts, 50/80 [62.5%] students, 46/85 [54.1%] patients), and 7.4% were neutral (1/104 [1.0%] experts, 10/80 [12.5%] students, 9/85 [10.6%] patients). Experts expressed significantly more positive attitudes than skepticism compared with students and patients. The differences were significant (χ²_3_=11.45, n=184, *P*=.01 [experts vs students]; χ²_3_=12.19, n=189, *P*=.01 [experts vs patients]), but not for students compared with patients.

Patients of the clinic expressed skepticism (15/41 [36.6%]), positive attitudes (18/41 [43.9%]), and neutral attitudes (4/41 [9.8%]; missing: 4/41 [9.8%]), while patients recruited via the internet answered skeptically (11/44 [25.0%]), positively (28/44 [63.6%]), and neutrally (5/44 [11.4%]). This difference was not significant (χ²_3_=6.80, n=85, *P*=.08).

The three groups differed in their opinion about whether e-mental health apps will gain importance in the future (*F*_2,263_=7.64, *P*=.001). The experts believed this (mean 1.08, SD 0.68, 95% CI 0.94-1.21), while students and patients did not share this attitude (students: mean 0.79, SD 0.85, 95% CI 0.60-0.98; patients: mean 0.60, SD 0.99, 95% CI 0.39-0.82). There was a significant difference between experts and patients (Dunnett-T3: *M_diff_* [mean difference]=0.48, SE 0.13, *P*=.001, 95% CI 0.17-0.78) and between experts and students (*M_diff_*=0.29, SE 0.12, *P*=.04, 95% CI 0.01-0.57) but not between students and patients (*M_diff_*=0.19, SE 0.14, *P*=.49, 95% CI –0.53 to 0.16).

**Table 4:** Results for the question, “What do you think in general of e-mental health apps?”

Response	Experts (n=104), n	Students (n=80), n	Patients (n=85), n	Total (N=269), n (%)
I think, there's great potential in the development.	22	15	16	53 (19.70)
I'm basically in favour of the development.	49	35	30	114 (42.38)
I'm in favour of the development of health apps, but not for mental disorders.	1	10	9	20 (7.43)
I am sceptical about the development.	25	16	24	65 (24.16)
I am concerned about the development.	6	4	2	12 (4.46)
Not answered	1	0	4	5 (1.86)

#### Qualitative Remarks

Many participants (n=199) took the opportunity to comment by answering the question: “Which positive aspects do you see regarding e-mental health apps?” These data were analyzed using content analysis by building inductive categories as described above. The most frequent category of answers was that an e-mental health app may deliver low-threshold access to psychotherapy. This view was shared by 70 participants (experts: 36, students: 21, patients: 13). Further, 53 statements expressed the belief that such an app improved everyday support (experts: 23, students: 9, patients: 21), and 50 remarks referred to improved self-management with such apps (experts: 23, students: 13, patients: 14). Finally, 23 statements mentioned the possibility of documentation/monitoring of therapeutic progress (experts: 7, students: 6, patients: 10). In particular, the experts believed that an e-mental health app had a good chance to improve psychoeducation (22 remarks in total; experts: 16, students: 5, patients: 1).

### Expectations

#### Quantitative Results

The highest rated expectation toward an ideal e-mental health app was “privacy should be respected” with a mean score of 1.85 (SD 0.59, 95% CI 1.78-1.93). The lowest rated item was “it should be changeable and adaptable by me” with a mean of 0.66 (SD 1.14, 95% CI 0.52-0.80). All results are presented in detail in Table 5.

By principal component analysis, three dimensions could be extracted from the initial eight items: transparency, costs, and design/customizability; these accounted for 55.1% of the variance (Table 6). A subsequent ANOVA showed that the groups differ in their expectations (*F*_2,261_=3.28, *P*=.04). The experts attached more importance to transparency than the patients (Scheffé: *M_diff_*=0.38, SE 0.15, *P*=.04, 95% CI 0.01-0.74), while the latter put more emphasis on costs compared to the experts (*M_diff_*=0.59, SE 0.14, *P*<.001, 95% CI 0.23-0.94) and design/customizability compared to the experts (*M_diff_*=0.45, SE 0.15, *P*=.01, 95% CI 0.09-0.81). All other comparisons were without significant differences.

**Table 5:** Results for the question, “What functions or properties would you expect in an ideal e-mental health app?” (for all: min=–2, max=2).

Item	Participants (N=269), n (%)	Mean (SD)	95% CI
It should be changeable and adaptable by me.	267 (99.26)	0.66 (1.14)	0.52-0.80
It should not cost much.	266 (98.88)	1.05 (1.06)	0.92-1.18
It should be covered by the health insurance.	266 (98.88)	0.67 (1.20)	0.52-0.81
The purpose of the exercises should be clear and concise.	267 (98.26)	1.61 (0.68)	1.53-1.69
It should be clear who designed the app.	268 (99.63)	1.04 (1.14)	0.90-1.18
There should be scientific evidence of efficacy.	268 (99.63)	1.21 (0.92)	1.10-1.32
The design should be appealing.	268 (99.63)	1.13 (0.91)	1.02-1.24
Privacy should be respected.	268 (99.63)	1.85 (0.59)	1.78-1.93

**Table 6:** Matrix of components after varimax-rotation (the rotation is converged in 5 iterations; method of extraction: main component analysis).

Component	Transparency	Costs	Design/customizability
It should be clear who designed the app.	0.72	–0.17	–0.08
There should be scientific evidence of efficacy.	0.70	0.03	0.03
Privacy should be respected.	0.62	0.16	0.10
The purpose of the exercises should be clear and concise.	0.54	0.40	0.12
It should be covered by the health insurance.	0.11	0.71	0.25
It should not cost much.	–0.05	0.79	–0.18
The design should be appealing.	0.42	0.26	–0.56
It should be changeable and adaptable by me.	0.25	0.18	0.77
Eigenvalue	2.18	1.22	1.01
Percentage of total variance	27.27	15.25	12.56
Total variance	—^a^	—^a^	55.09^b^

^a^Not applicable.

^b^Deviations due to rounding.

#### Qualitative Remarks

The quantitative results are supported by a closer look at the statements made in open-text fields: Of the 19 statements in total, those referring to usability and customizability were nearly all expressed by students and patients (usability: experts: 1, students: 4, patients: 1; customizability: experts: 0, students: 0, patients: 2). The remarks of the experts in contrast referred to an improved risk management and possibilities for the patients to get in contact with a psychotherapist (risk management: experts: 3, students: 1, patients: 1; contact: experts: 2, students: 0, patients: 1).

### Risks

All groups had concerns regarding the lack of data protection (mean 0.85, SD 1.09, 95% CI 0.72-0.98). All results are presented in Table 7. An ANOVA showed that there were no differences between the groups except in one item: “The exercises don’t help.” Students and patients showed significantly more concerns than experts (*F*_2,261_=6.03, *P*=.003; students vs experts: *M_diff_*=0.48, SE 0.15, *P*=.004, 95% CI 0.13-0.83; patients vs experts: *M_diff_*=0.42, SE 0.16, *P*=.003, 95% CI 0.04-0.81).

The mean total score of all four risks was 0.11 (SD 0.74, 95% CI 0.02-0.19). There was no significant result after comparison of the three groups via an ANOVA (*F*_2,261_=0.28, *P*=.76).

**Table 7:** Results for the question, “What risks do you see in e-mental health apps?” (for all: min=–2, max=2).

Item		Participants, n (%)	Mean (SD)	95% CI
**The exercises don’t help.**				
	Experts	103 (99.04)	–0.01 (1.04)	–0.21 to 0.19
	Students	79 (98.75)	0.47 (0.92)	0.26 to 0.67
	Patients	82 (96.47)	0.41 (1.12)	0.17 to 0.66
	Total	264 (98.14)	0.27 (1.05)	0.14 to 0.39
**The exercises are harmful for the health.**				
	Experts	103 (99.04)	–0.50 (0.96)	–0.68 to –0.31
	Students	79 (98.75)	–0.46 (0.95)	–0.67 to –0.24
	Patients	81 (95.29)	–0.47 (1.21)	–0.74 to –0.20
	Total	263 (97.77)	–0.48 (1.03)	–0.60 to –0.35
**By using such apps social contacts get lost.**				
	Experts	104 (100.00)	–0.16 (1.32)	–0.42 to 0.09
	Students	79 (98.75)	–0.25 (1.21)	–0.53 to 0.02
	Patients	83 (97.65)	–0.31 (1.42)	–0.62 to 0.00
	Total	266 (98.88)	–0.24 (1.32)	–0.40 to –0.08
**The data are not protected.**				
	Experts	104 (100.00)	0.91 (1.04)	0.71 to 1.12
	Students	79 (98.75)	0.73 (1.11)	0.49 to 0.98
	Patients	83 (97.65)	0.88 (1.13)	0.63 to 1.13
	Total	266 (98.88)	0.85 (1.09)	0.72 to 0.98

#### Qualitative Remarks

The item “What would stop you from using an e-mental health app?” received 69 comments; all three groups expressed the fear that the development of e-mental health apps could promote a tendency toward the “transparent patient” caused by a missing protection of privacy. Missing scientific background of an e-mental health app was named as the second most frequent barrier (31 comments) followed by the fear of replacement of real-life psychotherapy (25 comments). Another question for specific risks demonstrated that some of the experts and patients (n=7 in total) expressed their concerns that an e-mental health app could become a substitute for real-life psychotherapy. Finally, 8 participants stated that the patient is left alone without a feedback by using e-mental health apps. Another participant, a patient, wrote: “One is reminded of the illness. Every day.”

### Possible Determinants of Attitudes, Expectations, and Risks

#### Age

There was no significant correlation between age and general attitudes toward e-mental health apps. We found a slight but nonsignificant negative correlation of general attitudes with the age of the experts (*r_s_*(103)=–0.17, *P*=.09; students: *r_s_*(80)=–0.16, *P*=.15; patients: *r_s_*(81)=–0.07, *P*=.53).

The expectation that e-mental health apps will gain importance in the future correlated negatively with the age of the patients (*r*=–0.31, *P*-.005; experts: *r*=–0.19, *P*=.05; students: *r*=–0.01, *P*=.95).

The expectations toward an ideal e-mental health app were not related to the age of the participants, except in the factor transparency in the group of the patients: The older the patient, the more emphasis he or she put on the opinion that an ideal e-mental health app should be transparent (ie, clear who designed the app, scientific evidence, etc) with a highly significant association (*r*=.35, *P*=.002; experts: *r*=–0.01, *P*=.88; students: *r*=–0.02, *P*=.87).

In the group of patients, age also correlated positively with the total risk score (*r*=0.22, *P*=.05; experts: *r*=0.17, *P*=.09; students: *r*=–0.03, *P*=.81).

#### Professional Experience of the Experts

The professional experience of the experts ranged between 1 and 33 years (physicians: mean 14, SD 10; psychological psychotherapists: mean 17, SD 10; psychological psychotherapists in training: mean 3, SD 2; nurses: mean 18, SD 10).

Regarding the attitudes toward e-mental health apps, there was a significant negative correlation with the professional experience of the experts (*r_s_*(94)=–0.23, *P*=.03). The longer a medical expert had already practiced his or her profession, the lower was the acceptance of e-mental health apps, in general. The opinion that e-mental health apps will gain importance in the future also correlated negatively with the years of professional experience (*r*=–0.23, *P*=.03).

The professional experience of the medical experts did not correlate with expectations toward an ideal e-mental health app (transparency: *r*=0.07, *P*=.50; costs: *r*=–0.18, *P*=.09; design/customizability: *r*=–0.13, *P*=.21).

There was a significant positive correlation between professional experience and the total risk score (*r*=0.21, *P*=.04).

#### Sex

We found no differences in sex regarding general attitudes (experts: *U*_48,55_=519.5, *P*=.57; students: *U*_27,53_=561.0, *P*=.10; patients: *U*_18,63_=519.5, *P*=.57) or the expectation of the role of e-mental health apps in the future (experts: *F*_1,101_=0.25, *P*=.62; students: *F*_1,78_=1.07, *P*=.30; patients: *F*_1,81_=1.39, *P*=.24).

## Discussion

### Principal Findings

This study investigated the attitudes, expectations, and concerns toward e-mental health apps of medical experts, students of medicine or psychology, and patients. A special interest was to explore the role of previous knowledge of and former experiences with e-mental health apps made by the participants. Do these factors help reduce reservations about the utility of these tools, and thus, in the long term, increase user engagement? The results showed that in spite of a moderate knowledge of e-mental health apps, there was very little experience with these apps, especially in the group of medical experts and students who will be the future experts. However, a distinct group of patients were already in touch with e-mental health apps; they were not patients of the clinics but patients who joined the study via the internet and had self-reported mental health problems. If present, the personal experiences were rated positively. Trial of an e-mental health app led to a decrease in the reservations about the issue. However, the number of those patients who had personal experiences was too low to allow for conclusions based on further statistics.

In general, the highest acceptance of the development of e-mental health apps was expressed by the medical experts. They more often expected a growing importance of the topic in the future than students and patients. They recognized that online interventions could reduce the threshold to psychotherapy and deliver potentially useful psychoeducation for patients.

When asked for the possible risks, all groups reported concerns regarding data security. The experts were less worried about a potential risk of ineffective interventions. This risk was considered more likely by students and patients. Current medical and psychological experts may trust the state of the development more than others. Yet, the results show that experts especially express their wish to be informed about scientific evidence, purpose, and the developers of apps, which was summarized in the factor “transparency” in this study. From the patient’s perspective, financial aspects and customizability were more relevant.

Regarding the topic e-mental health apps, which is very close to technical innovations, in general, one could expect a strong moderating role of the factor age. In fact, March et al [18] showed earlier that this is only the case for therapist-assisted e-mental health services, but not for self-help interventions. In our results, age, as a determinant for expectations or perceived risks, was only found in the group of the patients (aged 18-77 years). In the group of the experts, the years of professional experience (range: 1-33 years) could explain more than age: The higher the experience, the lower was the acceptance of e-mental health apps, in general, and the expectation that e-mental health apps will gain importance in the future.

### Implications and Recommendations

The implications of the results should be reflected separately for the three groups investigated.

A closer look at the attitudes of medical experts reveals that psychotherapists, especially in the surveys of Schröder et al [25] and Tonn et al [24], expressed more critical opinions (in both cases, toward internet interventions, in general) than they did in our study. However, the often-reported fear of therapists of being replaced by internet-based therapy [8] could not be confirmed in our study. Maybe our results help explain the concerns in a more subtle way. Looking at the qualitative remarks of the experts, many therapists do not fear losing their patients to the internet, but the therapeutic relationship could be missing for the patients, which is not the same. The importance of the therapeutic relationship is well investigated and documented [35-37], and the focus of further research should lie on the question of how responsibilities of experts change with parallel therapeutic offers via an app. Some of their patients may rely on possible untrustworthy content of semiprofessional apps, which may be potentially harmful [23]. In this context, we recommend increasing the awareness of changing professional roles by promoting vivid discussions about e-mental health topics via conferences. As reported in the introduction, there is a current lack of exchange on the topic [20], especially among experienced by senior medical experts. Regarding these new responsibilities due to technical innovations, experts should have access to guidelines in order to assess the quality of e-mental health apps, as the German Federal Chamber of Psychotherapists already requires [38].

The implications have a direct impact on the role of the future experts—the students of medicine or psychology. Although some of them are already in touch with e-mental health apps for their own use, as indicated by studies conducted in the United States [29], students expressed little knowledge and experiences in our study. Regarding the still-growing market of e-mental health apps, assessing the seriousness and scientific evidence of these apps should become part of the curricula. There are only a few years left until today’s students will have to be able to make reasoned decisions and assess the circumstances when confronted with patients who want to use an e-mental health app or already did and were confused by them.

In this study, patients were the group with the largest amount of knowledge and the widest experience with e-mental health apps. There is a certain group of patients who act autonomously and care for its own recovery and self-management. This goes along with the observation that by being digital, patients increasingly gain independence [39]. Similar to our work, Schröder et al [25] reported that patients in nonclinical settings showed more positive attitudes toward internet interventions than clinical patients [25]. In our study, we found a similar, but not statistically significant, tendency. Recent results show that patients with negative care experiences tend to prefer eHealth services; this is especially true for those patients with a lower educational level [17]. March et al [18] reported that the willingness to use internet-based mental health services depends on the individual diagnosis. Patients with depression are more likely to prefer these services than patients with anxiety [18].

Based on these results, we recommend a personalized approach. Patients in clinical settings may need encouragement to try an established, validated e-mental health app (eg, for self-management of symptoms during after-care). Patients with negative prior care experiences or fear of clinical settings are difficult to be reached directly. For them, established standards for the development of e-mental health apps are highly needed. These implications support the recommendations referring to the expanded responsibilities of the experts and the future experts, as discussed above.

### Study Limitations

As part of our study, we presented the participants with a short list of current programs and apps. This list could be complemented manually by the participants, and many of them took advantage of this possibility. Nevertheless, the development of e-mental health apps is a rapidly changing market, and the results may soon become obsolete.

Furthermore, the willingness to respond to a questionnaire with a focus on e-mental health apps depends to a high degree on an affinity to the internet or technology-related topics. This may be one reason for the positive responses toward the future of e-mental health apps. In particular, patients in the clinic who refused to take part in the study told us that they were not used to smartphone technology at all and that they did not feel competent enough to complete the questionnaire. A similar effect might exist in case of the experts contacted via email.

Another limitation lies within the group of the patients. Less than one half of these patients could be recruited in the Department of Internal Medicine of the University Hospital of Heidelberg. The remainder was found in self-help groups and forums on the internet and they were, not surprisingly, more in touch with the e-mental health app topic than the patients of the clinic. A bigger survey with a more representative sample of patients, also including outpatient services, would reveal a broader insight into the perspective of the patients. Further, some of the online patients may be medical experts or students as well, which was not asked explicitly.

We did not ask for the type of mental disorder of the patients, because patients of the psychosomatic departments often suffer from somatic diseases, which are accompanied by mental strains. Asking for a specific diagnosis would have raised the threshold for taking part in this survey. Further studies should focus on a more differentiated picture of the different types of mental disorders.

Finally, more research is necessary on the role of previous experiences with health care providers and the specific diagnosis of the patients as determinants of attitudes toward e-mental health apps.

### Conclusions

This study revealed a lack of knowledge and experience of e-mental health apps in experts, students of medicine and psychology, as well as in patients. Even though some concerns were expressed regarding the potential negligence of private data protection, the attitudes and expectations of the target groups were rather positive. This was associated with a younger age of the patients and less professional experience of the medical experts. In consideration of a growing market with professional and semi-professional offers in the app stores, a deeper understanding and awareness of experts and students is an urgent necessity.

## Abbreviations

ANOVA: analysis of variance
eHealth: electronic health
e-mental: electronic mental
SELFPASS: Self-administered Psycho Therapy SystemS
